# Supplementary material for: The Interaction Effect of Anti-RgpA and Anti-PPAD Antibody Titers: An Indicator for Rheumatoid Arthritis Diagnosis
Source: J Clin Med. 2023 Apr 21;12(8):3027. doi: 10.3390/jcm12083027 (PMC10144073; doi:10.3390/jcm12083027)
Supplement: Supplementary file 1 [file jcm-12-03027-s001.zip › Table S1.pdf]

Table S1. Comparison of Anti-RgpA, Anti-PPAD and Anti-PPAD/RgpA double positive with periodontitis markers stratified by presence or absence of RA.

Variable	AR		Control		AR		Control		AR		Control		AR		Control	
	RgpA				RgpA				PPAD				PPAD			
	<Q2	>Q2	<Q2	>Q2	<Q2	>Q2	<Q2	>Q2	<Q2	>Q2	<Q2	>Q2	<Q2	>Q2	<Q2	>Q2
**Number of Teeth**																
Median	19	21	25	26	20	21	25	25	20	19	25	25	20	19	25	25
(IQR)	(12-25)	(13-25)	(21-28)	(20-28)	(12-25)	(15-25)	(21-28)	(20-28)	(13-25)	(12-25)	(20-28)	(21-28)	(13-25)	(11-25)	(21-28)	(21-28)
**Plaque Index%**																
Median	61	71	53	51	64	73	54	49	64	71	57	27	69	66	54	46
(IQR)	(18-81)	(41-87)	(36-66)	(34-75)	(33-82)	(41-89)	(38-68)	(23-77)	(33-82)	(36-88)	(42-61)	(28-66)	(40-84)	(33-88)	(4-67)	(27-71)
**Gingival Index%**																
Median	44	52	32	29	48	53	32	16	48	50	33	31	52	48	33	3
(IQR)	(7-73)	(1-74)	(23-48)	(15-57)	13-73	4-73	21-51	9-49	10-73	10-79	17-51	17-52	10-71	10-81	18-49	16-57
**Bleeding on probing%**																
Median	36	**49****	40	37	41	**55****	40	32	44	47	40	39	46	49	40	39
(IQR)	(16-58)	**(32-68)**	(27-50)	(26-54)	2-58	**31-59**	27-50	8-54	26-63	20-63	27-55	24-50	26-62	20-67	27-54	28-51
**Pocket Depth mm**																
Median	4	4	4	4	4	4	4	4	4	4	4	4	4	4	4	4
(IQR)	1-4.1	1-4.2	4-4.3	1-4.3	1-4.1	1-4.2	4-4.3	1-4.2	1-4.2	1-4.1	1-4.2	1-4.3	1-4.2	1-4.1	1-4.2	4-4.3
**CAL mm**																
Median	2.6	2.8	2.3	2.6	2.7	2.7	2.3	**2.7¨****	2.8	2.7	2.4	2.3	2.8	2.7	2.4	2.3
(IQR)	2.2-3.3	2.3-3.3	2-2.8	2-3.1	2.2-3-3	2.2-3-3	1.9-2.9	**2.4-3**	2.3-3.3	2.3-3.5	2-3	1.9-2.8	2.4-3.3	2.2-3.5	2-3.0	1.9-3
**CAL (%)**																
Median	85	90	46	**56****	87	**90****	52	50	86	87	53	45	87	87	51	50
(IQR)	54-98	67-97	32-70	**39-84**	60-98	**65-99**	32-77	48-70	54-97	65-97	34-78	31-70	60-97	63-96	34-72	35-82

** $p<0.05$. RIQ= Rango Intercuartil; RgpA= Arg-gingipaína A; PPAD= peptidil arginina deiminasa de *P. gingivalis*; BOP=Sangrado al sondaje; CAL=Perdida de inserción Clínica.
