# Supplementary material for: The Interaction Effect of Anti-RgpA and Anti-PPAD Antibody Titers: An Indicator for Rheumatoid Arthritis Diagnosis
Source: J Clin Med. 2023 Apr 21;12(8):3027. doi: 10.3390/jcm12083027 (PMC10144073; doi:10.3390/jcm12083027)
Supplement: Supplementary file 1 [file jcm-12-03027-s001.zip › Table S2.pdf]

Table S2. Comparison of levels of Anti-RgpA, Anti-PPAD and AntiPPAD-RgpA double positive with periodontitis severity and RA activity.

Variable	RA Periodontitis +	RA Periodontitis -	RA Severity +	RA Severity -	RA Activity +	RA Activity -
**RgpA Q2**						
**<Q2**	44 (80)	11(20)	43 (78)	12(22)	16(29)	39(71)
**≥Q2**	75(85)	13(15)	75 (85)	13(15)	31(35)	57(65)
**RgpA Q1**						
**<Q1**	79(81)	18(19)	78 (80)	19 (20)	28(29)	69(72)
**>Q1**	40(87)	6(13)	40 (87)	6 (13)	19(9)	27(91)
**PPAD**						
**<Q2**	53(89)	6(11)	52(88)	7(12)	18(31)	41(69)
**>Q2**	66(79)	18(21)	66(79)	18 (21)	29(35)	55(65)
**PPAD**						
**<Q1**	**79(89)****	10(11)	**78 (88) ****	11(12)	27(30)	62(70)
**>Q1**	40(74)	14 (26)	40(64)	14(36)	20(37)	34(63)
**PPAD/Rg**						
**pAQ2**	95 (85)	17 (15)	94 (84)	18 (26)	18 (30)	42(70)
**Positive**	24 (77)	7 (23)	24 (77)	7 (33)	29 (35)	54(65)
**Negative**						
**PPAD/Rg**						
**pAQ1**	105(84)	20 (16)	104(83)	21(17)	29(30)	70(70)

**Positive negative**	14 (78)	4 (22)	14(78)	4 (28)	18(41)	26 (59)

** Significant difference ($p<0.05$). RA= Rheumatoid arthritis. RgpA= arginine-gingipain; PPAD= Pg-peptidyl arginine deiminase.
