# Supplementary material for: The Interaction Effect of Anti-RgpA and Anti-PPAD Antibody Titers: An Indicator for Rheumatoid Arthritis Diagnosis
Source: J Clin Med. 2023 Apr 21;12(8):3027. doi: 10.3390/jcm12083027 (PMC10144073; doi:10.3390/jcm12083027)
Supplement: Supplementary file 1 [file jcm-12-03027-s001.zip › Table S3.pdf]

**Table S3**. The operational capacity of anti-RgpA, anti-PPAD, and RgpA-PPAD diagnostic tests.

| Anticuerpos | n | Sensibilidad | Especificidad | VPP | VPN | Área bajo la curva ROC |
|---|---|---|---|---|---|---|
| **Anti-RgpA** | 255 | 32% | 87.4% | 76.7% | 50% | 0.60 |
| **Anti-PPAD** | 255 | 38% | 69% | 61.4% | 46.4% | 0.53 |
| **RgpAQ1-PPADQ2** | 255 | 23.1% | 93.7% | 82.5% | 48.6% | 0.58 |
| **RgpAQ1-PPADQ1** | 255 | 12.6% | 95.2% | 78.3% | 45.9% | 0.54 |
